# Digital Health Applications (DiGAs) on a Fast Track: Insights From a Data-Driven Analysis of Prescribable Digital Therapeutics in Germany From 2020 to Mid-2024

**DOI:** 10.2196/59013

**Published:** 2024-08-29

**Authors:** Moritz Goeldner, Sara Gehder

**Affiliations:** 1 Working Group for Data-Driven Innovation Hamburg University of Technology Hamburg Germany

**Keywords:** digital health application, DiGA, data-driven analysis, clinical evidence, health economics, positive care effect, medical benefit, patient-relevant structural and procedural improvements, pSVV, digital health care act

## Abstract

**Background:**

This study aimed to analyze the rapidly evolving ecosystem of digital health applications (*Digitale Gesundheitsanwendung*; DiGAs) in Germany, spurred by the 2019 Digital Healthcare Act. With over 73 million people in Germany now having access to DiGAs, these prescribable digital health apps and web-based applications represent a substantial stride in health care modernization, supporting both patients and health care providers with digital solutions for disease management and care improvement.

**Objective:**

Through a data-driven approach, this research aimed to unpack the complexities of DiGA market dynamics, economic factors, and clinical evidence, offering insights into their impact over the past years.

**Methods:**

The analysis draws from a range of public data sources, including the DiGA directory, statutory health insurance reports, app store feedback, and clinical study results.

**Results:**

As of July 1, 2024, there are 56 DiGAs listed by the Federal Institute for Drugs and Medical Devices (Bundesinstitut für Arzneimittel und Medizinprodukte), divided into 35 permanently and 21 preliminarily listed applications. Our findings reveal that a majority of DiGAs extend beyond the intended 1-year period to achieve permanent listing, reflecting the extensive effort required to demonstrate clinical efficacy. Economic analysis uncovered a dynamic pricing landscape, with initial prices ranging from approximately €200 to €700 (€1=US $1.07), averaging at a median of €514 for a 3-month DiGA prescription. Following negotiations or arbitration board decisions, prices typically see a 50% reduction, settling at a median of €221. Prescription data offer valuable insights into DiGA acceptance, with total prescriptions jumping from around 41,000 in the first period to 209,000 in the latest reporting period. The analysis of the top 15 DiGAs, representing 82% of the total prescriptions, shows that these best-performing apps receive from a minimum of 8 to a maximum of 77 daily prescriptions, with native apps and early market entrants achieving higher rates. Clinical evidence from all 35 permanently listed DiGAs indicates a uniform preference for randomized controlled trials to validate primary end points, with no noteworthy use of alternative study designs encouraged in the Digital Healthcare Act and related regulations. Moreover, all evaluated DiGAs focused on medical benefits, with health status improvement as a key end point, suggesting an underuse of patient-relevant structural and procedural improvement in demonstrating health care impact.

**Conclusions:**

This study highlights the growth and challenges within the DiGA sector, suggesting areas for future research, such as the exploration of new study designs and the potential impact of patient-relevant structural and procedural improvements. For DiGA manufacturers, the strategic advantage of early market entry is emphasized. Overall, this paper underscores the evolving landscape of digital health, advocating for a nuanced understanding of digital health technology integration in Germany and beyond.

## Introduction

### Background

While Germany continues to navigate the complex challenges of digital transformation within its health care system, the reimbursement of digital medical devices by statutory health insurance represents a pivotal milestone in this journey. The 2019 Digital Healthcare Act (Digitale-Versorgung-Gesetz) marks a substantial legislative milestone, incorporating digital health applications (German: *Digitale Gesundheitsanwendung*; DiGA) into the benefits catalog of statutory health insurance in Germany [1-4]. Since September 2020, the first DiGAs have been officially listed in the DiGA directory of the Federal Institute for Drugs and Medical Devices (German: Bundesinstitut für Arzneimittel und Medizinprodukte; BfArM), thereby becoming accessible to more than 73 million individuals in Germany. DiGAs are digital medical devices of risk classes I and IIa, or IIb (a recent regulatory update—the Digital Law—has made class IIb medical devices eligible for DiGA listing, albeit imposing more stringent criteria for demonstrating evidence and proving the positive care effect) that are primarily based on digital technologies and aim at assisting both insured individuals and health care professionals by enhancing the detection, monitoring, treatment, or alleviation of diseases, as well as the identification of, treatment of, alleviation of, or compensation for injuries or disabilities. DiGAs listed in the DiGA directory may be prescribed by physicians and psychotherapists or directly provided by the statutory health insurances under certain requirements [5,6].

Compared with existing approval procedures in health care, the legislator has integrated 3 regulatory innovations. First, a 12-month trial phase allows DiGA manufacturers to initially introduce their DiGA to the market on a provisional basis and subsequently provide the final proof of effectiveness [6]. During this phase, the price for using the DiGA can be set by the manufacturer within defined maximum limits. Second, the approval was designed as a fast-track-procedure, meaning the BfArM’s review of the application takes a maximum of 3 months. Third, the concept of “therapeutic benefit,” crucial in drug approval, has been expanded to the more comprehensive concept of a “positive care effect” (German: *Positiver Versorgungseffekt*; pVE). This can be demonstrated either by proving a “medical benefit” (German: *medizinischer Nutzen*), that is, an improvement in health status or enhancement of quality of life, or a “patient-relevant structural and procedural improvement” (German: *patientenrelevante Struktur- und Verfahrensverbesserung*; pSVV) focusing on optimizing the interaction between patients and health care providers as well as supporting the patient [6-8]. To prove the positive care effect, each DiGA requires conducting a clinical trial within Germany. In addition, a preliminary study demonstrating the DiGA’s basic features is required before preliminary listing as well.

After almost 4 years into the implementation of the DiGA framework, we seek to investigate the evolving landscape of the DiGA market. Other European countries, such as France and Belgium, are adopting comparable approaches for digital medical device reimbursement [9,10], though they have not reached the same scale yet. Thus, our primary aim was to unpack the dynamics that have shaped this market in Germany, quantifying the economic aspects of DiGAs, and identifying the characteristics of successful DiGA implementations based on the data available. Moreover, this study aimed to provide a comprehensive understanding of the clinical evidence surrounding DiGA that has been generated and was assessed by BfArM.

### Objectives

To achieve these objectives, we adopt a data-driven approach, leveraging publicly available data sets to craft a coherent and detailed picture of the DiGA ecosystem. This methodology allows us to explore various facets of the DiGA landscape, from market trends and economic impact to clinical effectiveness and regulatory outcomes. Through this analysis, we aimed to uncover insights into the factors that contribute to the success of DiGA, the challenges they face in the market, and the broader implications for health care professionals, policy makers, and patients within the (digital) health care system.

## Methods

To provide a comprehensive analysis of the DiGA ecosystem in Germany, our study leveraged publicly available data sources. The following data sources were obtained during the analysis.

### DiGA Directory Data

Access to this data was facilitated via the DiGA application programming interface provided by BfArM, enabling up-to-date insights into the range and characteristics of each individual DiGA ever listed. The data were accessed using a custom-developed script using the cloud–based integrated development environment Replit.

### Annual Reports of Statutory Health Insurance Funds

We analyzed 3 annual reports on DiGAs from the National Association of Statutory Health Insurance Funds (German: GKV-SV), which provided valuable information on the adoption rates and prescription trends for DiGAs [11-13]. It is important to consider that this data set does not include privately insured patients who use DiGAs, potentially underrepresenting total use. In Germany, about 10% of the population is privately insured [14].

### App Store Ratings

To gauge user reception and satisfaction, we accessed app rating data from the Apple App Store (Apple, Inc) [15-17]. This was achieved through an application programming interface and a custom-developed Google Sheets (Alphabet, Inc) script, allowing for the automated collection of ratings data. Similarly, app ratings from the Google Play Store were manually accessed to complement the data gathered from the Apple App Store, ensuring a comprehensive view of user feedback across major mobile platforms [18].

### Accessing Clinical Studies

We conducted manual searches for all studies pertaining to permanently listed DiGAs that were identified as relevant for the “assessment decision by the BfArM” according to the DiGA directory. These studies were registered either in the German Clinical Trials Register, ClinicalTrials.gov (maintained by the National Library of Medicine at the National Institutes of Health), or the International Standard Randomised Controlled Trial Number registry (maintained by BioMed Central, part of Springer Nature). In addition, respective publications, study reports and protocols for each study were reviewed to assess the clinical evidence base supporting the use of these DiGAs. Our analysis encompassed a thorough review of study designs, measuring instruments, and dropout rates, analyzing the ratio of observation duration, sample size, and the length of the trial phase, and comparing age and gender of the participants with demographic data.

To ensure a uniform perspective across the collected data, our analysis specifically used information from the DiGA directory as of July 1, 2024. This approach provided us with a comprehensive data set encompassing 3 full years, in addition to approximately 3 months and a few days, since the first DiGA was introduced in late September 2020 and 6 months in 2024. It is important to note, however, that the data from the GKV-SV, particularly concerning the number of prescriptions, were segmented into 3 terms, with each term spanning from October to September of the following year. This discrepancy in the data collection periods has been carefully addressed in our analysis to ensure clarity and accuracy in our findings.

All data were consolidated into a single database. Basic calculations were performed using SPSS Statistics (version 29; IBM, Corp), and visualizations were created with flourish.studio (Canva UK Operations Ltd) and ThinkCell (think-cell Software GmbH). Given the nascent state of the DiGA market and the limited number of apps available, our study did not use statistical analysis. The market’s current size does not support such an approach; however, this is anticipated to change as the market matures. Consequently, our results are primarily descriptive, offering insights into the present landscape of DiGAs, their use, user feedback, and the clinical evidence supporting their efficacy and utility.

### Ethical Considerations

Ethics approval does not apply to this study, as we evaluated publicly available, deidentified data on DiGA prescriptions [11-13] and did not involve any human participants during data collection.

## Results

### Descriptive Data on DiGAs

**Figure 1:**
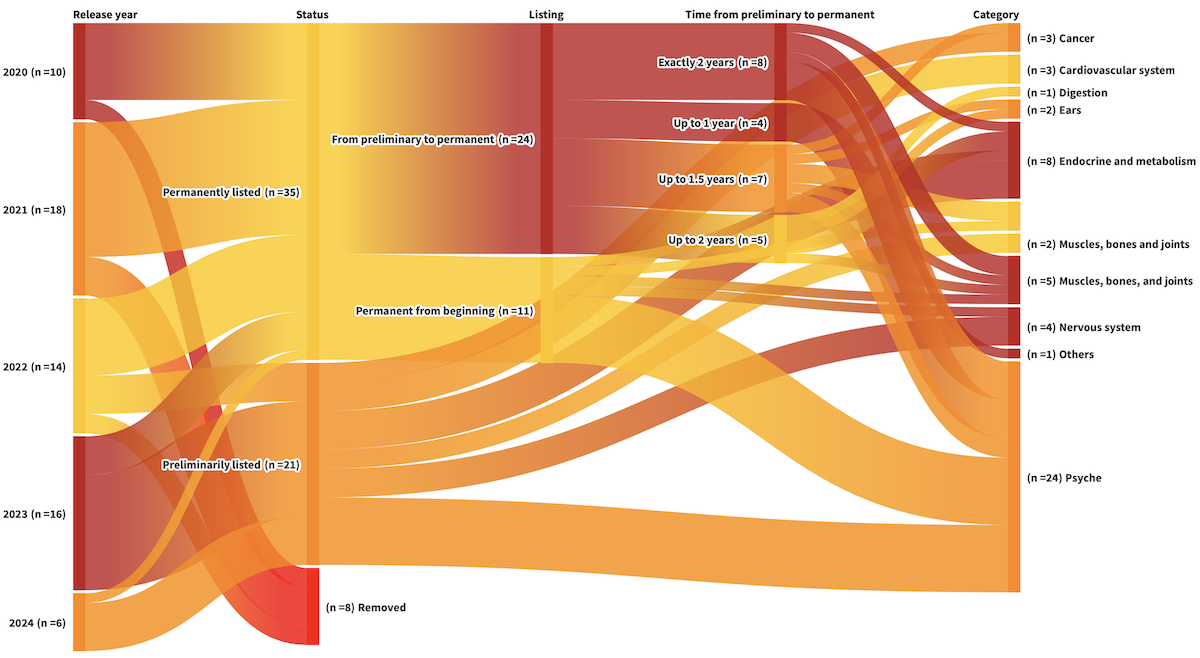
Overview of digital health application (Digitale Gesundheitsanwendung; DiGA) categories and preliminary or permanent listings. Only 1 therapeutic category is indicated for each DiGA.

As of July 1, 2024, a total of 64 DiGAs have been approved by the BfArM. Currently, 35 DiGAs are permanently listed, 21 are preliminarily listed (still required to submit clinical evidence for permanent listing), and 8 DiGAs have been removed from the directory. Among the 35 permanently listed DiGAs, 11 (31%) were initially approved for permanent listing, whereas 24 (69%) initially received preliminary approval and were later granted permanent listing. While the regulator allows up to 1 year for this process, an extension to 2 years is permissible (Figure 1).

Our analysis indicated that most DiGAs necessitate considerably longer than 1 year to complete this transition, with one-third precisely requiring 2 years (including the period for BfArM to assess the submitted data). Moving forward, we have excluded the 8 removed DiGAs from all subsequent analyses, concentrating on the 56 DiGAs that remained accessible.

The therapeutic areas for DiGAs span 12 categories as defined by the BfArM, with the largest being the *psyche* category. This category includes 26 DiGAs designed to address various psychological conditions, predominantly through cognitive behavioral therapy. The *endocrine and metabolism* category follows with 8 DiGAs. The *muscles, bones, and joints* category is represented by 7 DiGAs, and *nervous system* with 4 DiGAs (notably, 5 DiGAs are linked to 2 categories each, resulting in a total of 61 categorizations).

The new concept of positive care effect is the pivotal point in the assessment of clinical evidence for DiGA. Despite the regulator introducing a new framework, the pSVV, its uptake remained modest. Among the 56 DiGAs, 55 (98%) had a medical benefit as primary end point, while only 1 (2%) DiGA (ProHerz, ProCarement GmbH) had a pSVV as primary end point. In addition, 10 (18%) DiGAs with a medical benefit as primary end point incorporated pSVV as an additional end point in their studies.

To qualify for DiGA listing, a product must be certified as a medical device. Among the 56 DiGAs, 32 (57%) were still recognized under the Medical Device Directive (MDD) as class I medical devices—a regulation that officially expired in May 2021, with a current transition period in effect. The remaining 24 (43%) DiGAs complied with the Medical Device Regulation (MDR), the updated European standardized regulation where the assessment process for digital medical devices has changed (refer to rule 11 of MDR), resulting in a more rigorous review of their risk classes [19]. Within this group, 21 (87%) were classified as class I medical devices, and 3 (13%) were class IIa medical devices, which necessitate certification from a notified body for approval. Until July 2024, no class IIb medical device was certified as a DiGA, because applications for DiGAs in this risk class only became possible after the new regulation took effect at the end of March 2024. While MDR certification certainly enhances the safety of (digital) medical devices by requiring premarket testing and continuous postmarket surveillance of medical devices, it also represents a significant financial and time-consuming burden, particularly when it comes to class IIa or IIb medical devices because notified bodies are a veritable bottleneck within this process [19,20]. Variations were observed in relation to the federal states hosting the headquarters of DiGA manufacturers. From Hamburg, 20 DiGAs (from 8 manufacturers) emerge, with an approximately equal distribution between MDR (11 DiGAs) and MDD (9 DiGAs) classifications. In Berlin, 11 DiGAs (from 6 manufacturers) fall exclusively under the category of MDD-regulated medical devices, with only 1 DiGA listed in 2024 being classified under the MDR. Such disparities are not evident in other federal states. While we found no reason for this discrepancy in our data, we assumed that the state authorities in the city-states of Berlin and Hamburg have different approaches to accepting class I MDR medical devices under rule 11 of MDR.

DiGAs are mandated to use digital technologies, which means they are not restricted to app formats alone. However, our observations show that 27 DiGAs, originating from 24 manufacturers, were available as native apps (unlike web applications, native apps are developed for a specific platform, such as the Apple App Store or the Google Play Store) in both the Apple App Store and Google Play Store. In addition, 10 DiGAs, from 6 manufacturers, provided access through both app and web application formats, whereas 19 DiGAs, from 7 manufacturers, were exclusively accessible via websites.

### Analysis of Initial and Final Prices

During the first year following the initial listing, whether preliminary or permanent, DiGA manufacturers have considerable freedom in setting prices, subject to certain criteria based on the demonstrated positive care effect (medical benefit, pSVV, or both), the *International Classification of Diseases, Tenth Edition* (*ICD-10*) category, and the pricing of existing DiGAs within that category [2,21]. Upon final listing—or after 12 months for DiGAs already permanently listed—price negotiations with the GKV-SV commence, with 3 meetings planned to reach an agreement. Should these negotiations fail, an arbitration board is engaged to reach a definitive decision. Thus, not all permanently listed DiGAs have an established final price yet. As of July 1, 2024, among the 35 permanently listed DiGAs in our data set, 30 (86%) had received their final pricing. These final prices were markedly less than the initial prices, spanning from 29% to 93% of the initial price, averaging at 50% of the initial amount.

DiGA manufacturers also have the option to adjust (ie, increase) the price once within the first year, although this occurs infrequently and was not further considered in our data set. In addition, our analysis did not account for varying prices between the initial and subsequent prescriptions, as this pricing strategy is seldom implemented.

**Figure 2:**
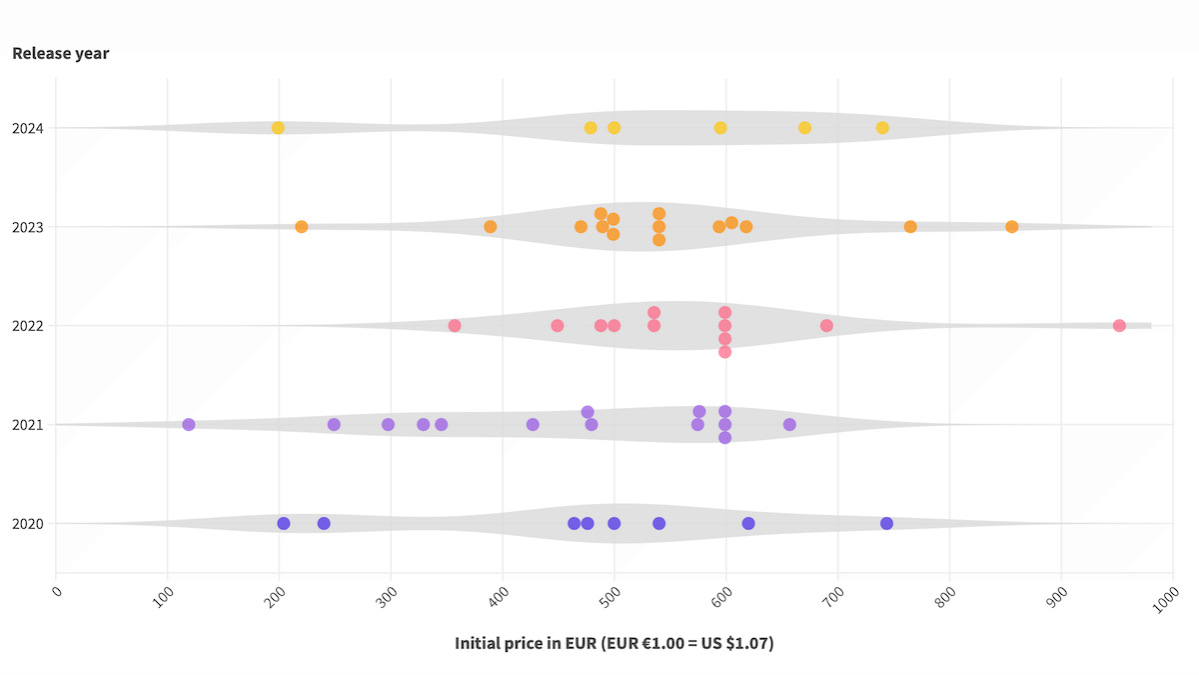
Initial prices of digital health applications (Digitale Gesundheitsanwendung [DiGA]; n=55) plotted per year of initial listing. One preliminary listed DiGA (levidex) with a lifetime license was excluded.

Typically, a DiGA is prescribed for 3 months of use. To date, only 1 preliminary listed DiGA, levidex, offered a lifetime license at a substantially higher cost of €2077.40 (€1=US $1.07). This DiGA will be excluded from the following analysis. The prices for the rest of the DiGAs were effective for a period of 3 months and ranged from approximately €200 to €700, with a median price of €535.49. One can observe that the initial pricing has trended upward over time (Figure 2). The median was €487 in the first year, increased to €567 in 2022, and adjusted to €547 in 2024 (Table 1).

**Table 1:** Initial and final prices of digital health applications (DiGAs) per release year. One preliminary listed DiGA (levidex) with a lifetime license was excluded.

Release year			Price (€^a^), mean (SD)		Price (€), median (IQR)
**Initial prices**					
	2020 (n=8)	473 (180)		488 (296-600)	
	2021 (n=14)	452 (162)		478 (321-599)	
	2022 (n=12)	575 (147)		567 (491-599)	
	2023 (n=15)	541 (147)		540 (488-605)	
	2024 (n=6)	531 (190)		547 (409-688)	
	All years (n=55)	514 (162)		535 (464-599)	
**Final prices**					
	2020 (n=8)	219 (16)		219 (209-229)	
	2021 (n=13)	219 (21)		223 (204-235)	
	2022 (n=6)	233 (9)		232 (224-238)	
	2023 (n=3)	228 (6)		232 (221-232)	
	All years (n=30)	221 (17)		224 (216-235)	

^a^€1=US $1.07.

**Figure 3:**
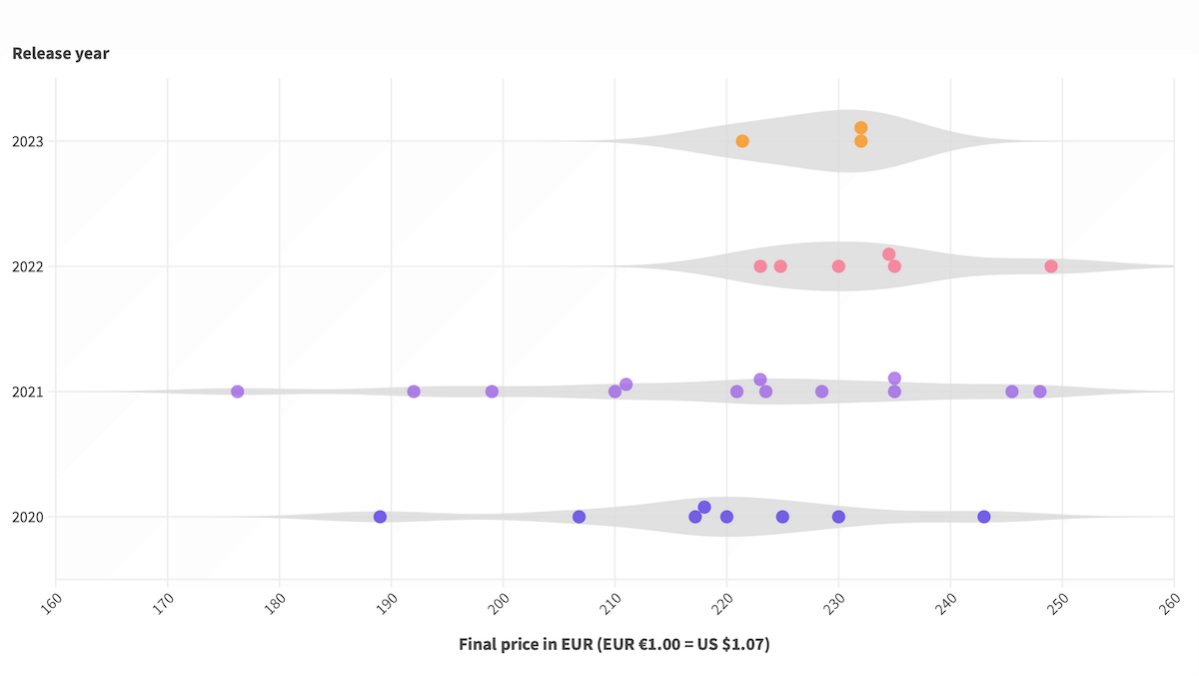
Final prices of digital health applications (Digitale Gesundheitsanwendung [DiGA]; n=30) plotted per year of initial listing.

Similarly, the prices negotiated or determined by the arbitration board have seen a gradual rise, moving from a median of €219 for DiGAs initially listed in 2020 to a median of €232 for those listed in 2023 for the first time (refer to Figure 3 for an overview). However, the data reveal low variance from the mean values of final prices.

**Figure 4:**
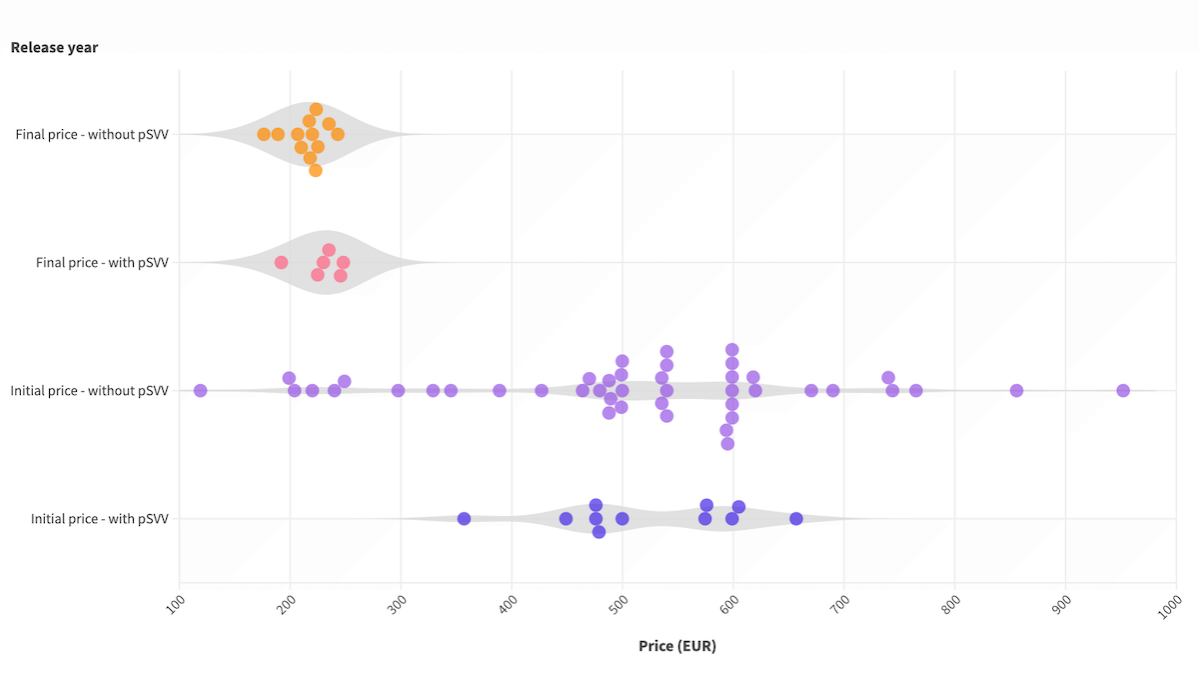
Initial prices of digital health applications (Digitale Gesundheitsanwendung [DiGA]; n=55) and final prices (n=30) with and without patient-relevant structural and procedural improvements (Patientenrelevante Struktur- und Verfahrensverbesserungen; pSVV).

In our subsequent analysis, we investigate whether the chosen positive care effect (ie, medical benefit or pSVV) impacts pricing. Initial prices showed a statistically insignificant difference (we used a Mann-Whitney *U* test as the data is not normally distributed; *P*=.69) in median values: €518 (IQR €398-€599) for DiGAs without pSVV compared with €479 (IQR €449-€576) for DiGAs with pSVV (Figure 4).

For the final prices, this insignificant difference was reversed; the median was €223 (IQR €212-€232) for DiGAs without pSVV and €232 (IQR €217-€246) for those that include a pSVV.

### Analysis of Prescriptions

Although prescription numbers for DiGAs likely offer some of the most compelling insights, these data are not publicly available for all DiGAs. The only official figures are annually published by the GKV-SV, which releases rounded prescription numbers. The total number of prescriptions was approximately 41,000 in the first reporting period (September 2020-September 2021), increased to 124,000 in the subsequent period (October 2021-September 2022), and reached 209,000 prescriptions in the latest period (October 2022-September 2023). Altogether, exactly 374,377 DiGA prescriptions were recorded from the initial availability of the first DiGA in late September 2020 until September 30, 2023. This total number also includes prescriptions of now delisted DiGAs.

However, although data on individual DiGA prescriptions were accessible for all DiGAs exceeding 100 total prescriptions in the first reporting period, this criterion tightened in later periods. In the subsequent period, only DiGAs with over 1000 total prescriptions throughout their life span were featured. The threshold was further elevated in the most recent report to include solely DiGAs with more than 5000 prescriptions over their life span. By the end of September 2023, 15 DiGAs surpassed the threshold of 5000 prescriptions over their lifetime. Hence, our subsequent analyses focused exclusively on these top 15 DiGAs. When available, data from 2021 and 2022 were also incorporated. To ensure consistency and comparability, we calculated the average daily prescriptions for these 15 DiGAs by taking the number of days listed in the directory from the first listing to the end of the reporting period of the GKV-SV at the end of the third quarter of 2023.

**Figure 5:**
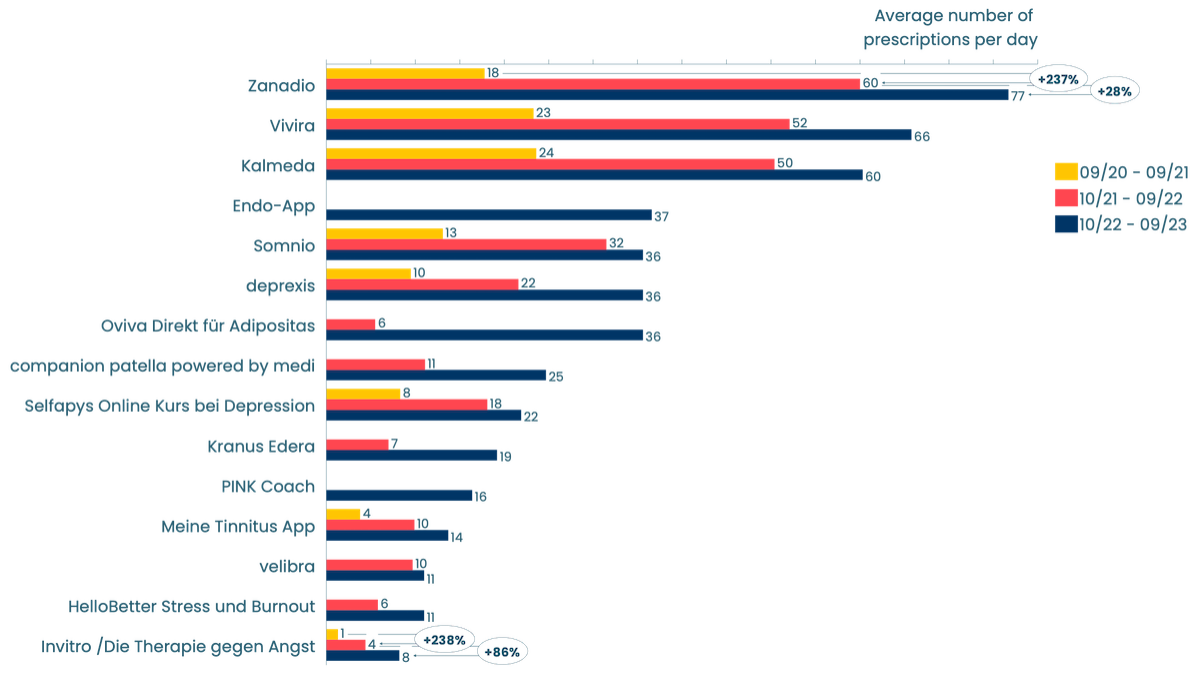
Average daily prescriptions per digital health application (Digitale Gesundheitsanwendung [DiGA]; n=15).

Within the top 15 DiGAs, we observed considerable variability in prescriptions, ranging from 77 per day for zanadio (Sidekick Health Germany GmbH) to 8 per day for Invirto (Sympatient GmbH). Among these top 15, 8 (53%) DiGAs were released in the first 12 months following the DiGA directory’s launch, 6 (40%) during the second period, and only 1 (7%) DiGA (Endo-App, Endo Health GmbH) during the latest reporting period by the GKV-SV. The average year-on-year growth for these apps across their individual first 2 reporting period was approximately 270%. However, between the second and third reporting periods, the growth rate for these apps decreased to 140% (Figure 5). For example, the growth rate for zanadio was 237% between the first two periods and 28% between the second two periods. Similarly, the growth rates for Invirto were 238% and 86%, respectively (Figure 5). In total, 308,000 prescriptions were made for these top 15 DiGAs, a substantial share (82%) of the total 374,377 prescriptions.

DiGAs are mandated to use digital technologies, which means they are not restricted to app formats alone. However, our observations showed that 27 DiGAs, originating from 24 manufacturers, were available as native apps in both the Apple App Store and Google Play Store. In addition, 10 DiGAs from 6 manufacturers provided access through both app and web-app formats, whereas 19 DiGAs from 7 manufacturers were exclusively accessible via websites. Within the top 15 DiGAs, it is notable that 9 DiGAs available solely as apps accounted for 210,000 prescriptions. In contrast, 2 DiGAs offering both app and web application formats garnered 46,000 prescriptions, and 4 DiGAs accessible only through web applications achieved 52,000 prescriptions.

While DiGA prescriptions are commonly associated with those issued by medical doctors or psychotherapists, there is an alternative pathway for patients to receive a DiGA directly from their statutory health insurance. Typically, health insurance providers may request proof that the patient has the condition targeted by the DiGA, though this is not an official requirement. In exploring the ratio of DiGAs distributed by health insurances versus total prescriptions, there are notable differences between the different DiGAs. Notably, Endo-App and Kranus Edera (Kranus Health GmbH), both categorized under *genitals, kidneys, and urinary tract*, lead health insurance distributions at 40% and 28%, respectively, followed by Oviva Direkt (Oviva AG) at 23%. Conversely, at the spectrum’s lower end, Vivira (ViViRA Health Lab GmbH) and Companion Patella (medi GmbH), focusing on muscles, bones, and joints, each saw only 5% of their distributions through statutory health insurance (Table 2).

**Table 2:** Digital health application (DiGA) prescriptions based on GKV-SV reporting [11-13].

DiGA name	Prescriptions from September 2020 to September 2021 (n=28,172), n (%)	Prescriptions from October 2021 to September 2022 (n=135,331), n (%)	Prescriptions from October 2022 to September 2023 (n=239,046), n (%)	Authorization by statutory health insurance (%)
Zanadio	6100 (21.65)	21,900 (16.18)	28,000 (11.71)	11
Vivira	8000 (28.4)	19,000 (14.04)	24,000 (10.04)	5
Kalmeda	8600 (30.53)	18,400 (13.6)	22,000 (9.2)	8
Deprexis	2100 (7.45)	7900 (5.84)	13,000 (5.44)	8
Endo-App	—^a^	—	13,000 (5.44)	40
Oviva Direkt	—	2000 (1.48)	13,000 (5.44)	23
Somnio	4500 (15.97)	11,500 (8.5)	13,000 (5.44)	11
Companion Patella	—	4000 (2.96)	9000 (3.76)	5
Selfapys Depression	2400 (8.52)	6600 (4.88)	8000 (3.35)	11
Kranus Edera	—	2000 (1.48)	7000 (2.93)	28
PINK Coach	—	—	6000 (2.51)	13
velibra	1400 (4.97)	3600 (2.66)	5000 (2.09)	8
HelloBetter Stress und Burnout	—	2000 (1.48)	4000 (1.67)	13
Meine Tinnitus App	—	2000 (1.48)	4000 (1.67)	6
Invitro	400 (1.42)	1600 (1.18)	3000 (1.18)	10

^a^Not available.

Finally, we also wanted to take into account the user’s reviews in the app stores as well as the information if the DiGA was the first within its *ICD-10* category on the market. Therefore, we can only consider the 11 (73%) out of the top 15 DiGAs that are listed as a native smartphone app in the 2 major app stores. App store reviews were collected from both, Apple App Store and Google Play Store and combined, taking the average review (on a 5-star scale) and the number of reviews into account. Again, we used average prescriptions per day to have better comparability.

**Figure 6:**
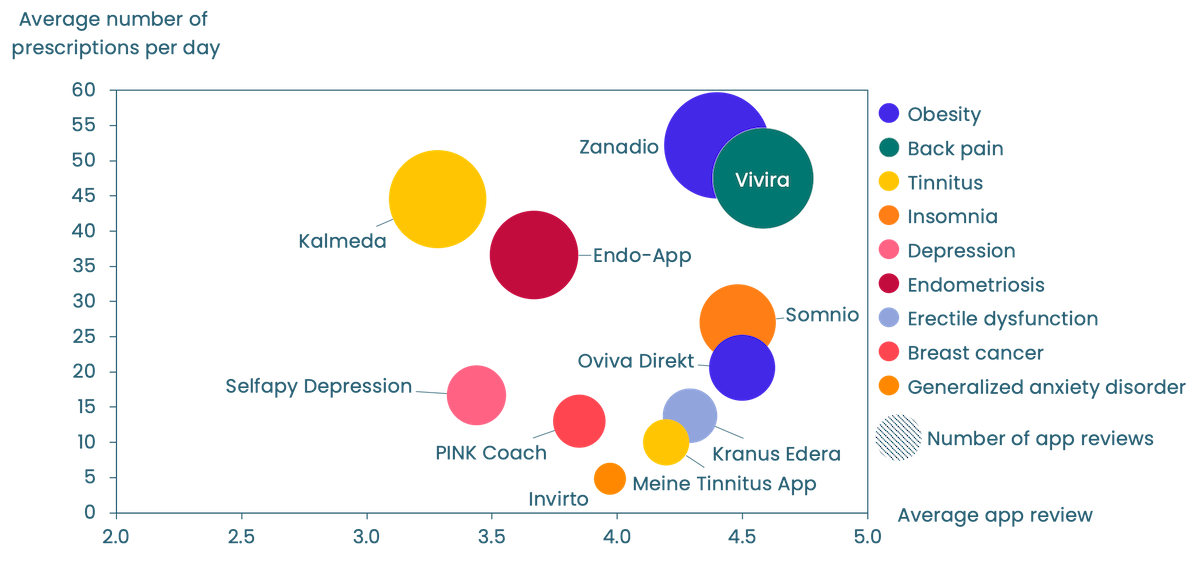
Average app reviews in app stores (x-axis) and daily prescriptions of digital health applications (Digitale Gesundheitsanwendung [DiGA]; y-axis) among the top 15 that are available as native apps. The size of the bubbles indicates the number of app reviews.

Unsurprisingly, it was observed that apps with a higher number of prescriptions tended to garner more reviews. Regarding the quality reflected in a 5-star rating system, Vivira, Oviva Direkt, somnio (mementor DE GmbH), and zanadio stood out with the highest reviews, scoring 4.6, 4.5, 4.5, and 4.4, respectively (Figure 6). On the lower spectrum, Kalmeda (3.3; Pohl-Boskamp Digital Health GmbH) and Selfapy (3.4; Selfapy GmbH; Selfapy provides a single app encompassing all their DiGAs, meaning reviews cannot be uniquely attributed to the app Selfapy Depression alone) were notable.

**Figure 7:**
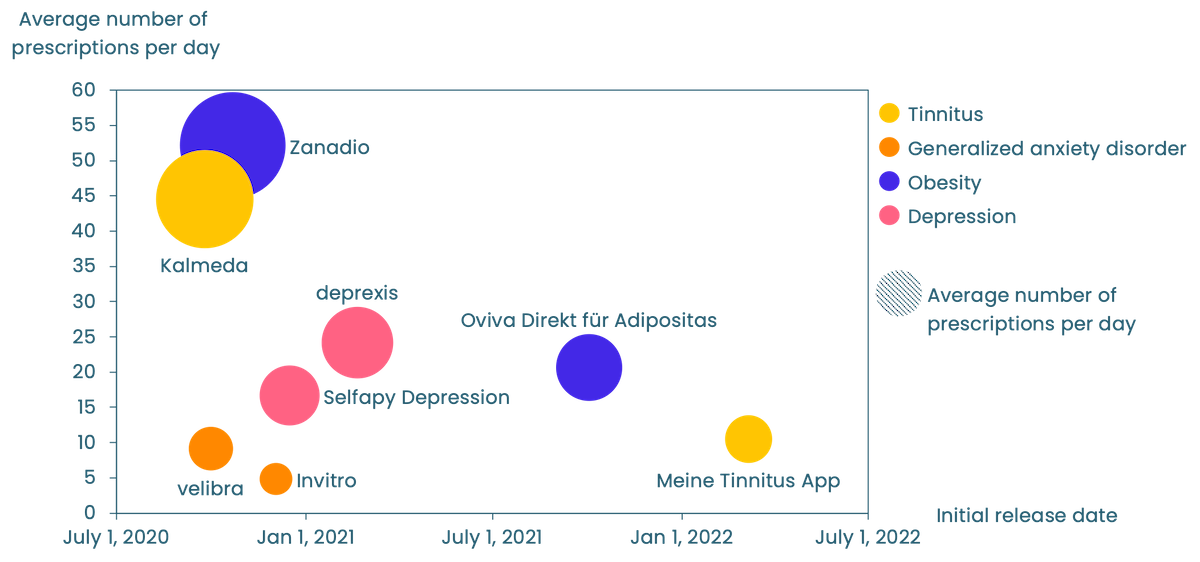
Average prescriptions per day (y-axis) and launch date (x-axis). The bubble size is proportionate to the average number of prescriptions per day.

In addition, we observed that DiGAs received more average prescriptions per day when they were first to the market than when they were second in various categories (Figure 7). This pattern was evident with zanadio leading ahead of Oviva Direkt, Kalmeda outpacing Meine Tinnitus App (BAYOOCARE GmbH), and—on a lower level—velibra (GAIA AG) ahead of Invirto. Only Deprexis (GAIA AG) performed slightly better than Selfapy Depression in terms of prescriptions, but both apps were—similar to velibra and Invirto—released only 2 months apart. While many factors might have contributed to this trend, the first-to-market advantage is worth noting, particularly if there is a long period between being first and second to market.

### Analysis of Clinical Trial Data

To demonstrate a pVE for permanent listing of a DiGA, manufacturers must conduct a clinical trial in Germany that shows one or more end points related to the positive care effect. In this section, we analyzed all permanently listed DiGAs in our sample (refer to Multimedia Appendix 1 for an overview).

Among the 35 DiGAs analyzed, all have proven at least 1 medical benefit, with each opting for *improvement of the state of health* as primary end point. Notably, 23 (66%) of these 35 DiGAs demonstrated this single positive care effect exclusively. Three more DiGAs demonstrated both, *improvement of the state of health* and *improvement in quality of life*. The remaining 9 DiGAs used pSVV as an additional end point in their studies.

In total, 5 DiGAs have evidenced a pSVV along with *improvement of the state of health*, covering aspects such as *health competence*, *reduction of therapy-related efforts and burdens on patients and their relatives*, *alignment of treatment with guidelines and recognized standards,* and *patient sovereignty*. Furthermore, 3 more DiGAs were able to show 2 medical benefits along with a pSVV (with all 3 opting for *patient sovereignty*), and finally, 1 DiGA demonstrated 2 medical benefits in conjunction with 2 additional pSVV (ie, *health competence* and *coping with illness-related difficulties in everyday life*).

Following the permanent listing of a DiGA, manufacturers are required to make available to the public, within 1 year, the study results demonstrating the pVE of the DiGA. As of July 2024, for all 35 permanently listed DiGAs in our data set, at least 1 registered clinical trial was mentioned in the “assessment decision of BfArM” of the DiGA directory. Furthermore, 1 of these 35 DiGAs (Deprexis) cited 2 clinical trials—hence, the subsequent analyses refer to a total of 36 registered trials.

All 36 trials were registered as randomized controlled trials. The study type was registered as interventional across all 36 studies. In 35 (97%) of the studies, the allocation of participants to either the control group or the intervention group was conducted through “parallel distribution,” while 1 (3%) study used a “factorial” design. Furthermore, 15 (42%) studies used masking, 19 (53%) explicitly did not, and 2 (6%) studies did not clarify their use of masking. Where implemented, masking usually concealed the group affiliation (ie, control or intervention) from either the outcome assessors, the statisticians, or both. Exceptionally, 1 study kept patients unaware of their group membership.

In the 36 conducted trials, 22 different primary end points (such as *depression symptoms*, *tinnitus exposure,* or *pain intensity*) were used. To evidence these primary end points, 29 different measurement instruments (mostly patient-reported outcome measures, but also measures such as weight or HbA_1C_) were used. Furthermore, 6 end points were used by >1 DiGA manufacturer—*depression symptoms*, *anxiety symptoms*, *pain intensity*, *body weight, reduction of disease-specific symptom severity,* and *tinnitus burden.*

Yet, these primary end points were measured differently by DiGA manufacturers—only within the end points *depression symptoms* and *tinnitus burden*, 2 psychometric tests for measuring the severity of depression (Beck Depression Inventory and Patient Health Questionnaire) and a Tinnitus Questionnaire (Mini-TF-12) were used 2 or 3 times, respectively. Thus, most DiGA manufacturers opted for different measures for the primary end point, even if the same end point has been previously assessed by another DiGA. For secondary end points, we see a variety of measures that were used across DiGA manufacturers.

**Figure 8:**
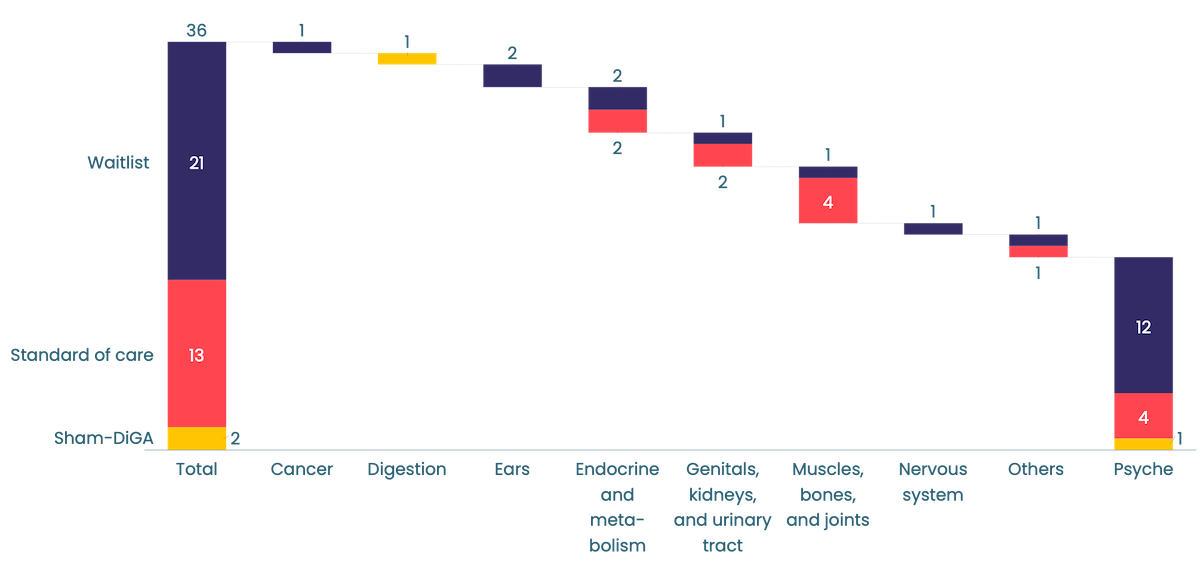
Control groups in clinical studies (n=36) per disease category. Two digital health applications (Digitale Gesundheitsanwendung; DiGAs) are associated with 2 disease categories. In this figure, both are located within the muscle, bones, and joints category; the second category was psyche (waitlist) and injuries (standard of care).

In total, 22 (61%) of the 36 studies used a waitlist control group, while 12 (33%) studies used *standard of care* as the control. Two DiGAs implemented control groups using apps with reduced functionality (sham DiGAs), featuring versions of the actual DiGA that provided only medically useful tips or survey instruments. There was no obvious pattern linking the design of these control groups with the indication area of the DiGA (Figure 8).

In total, 30 (86%) out of the 35 permanently listed DiGAs recommend a use duration of 3 months. Furthermore, 20 (57%) of these DiGAs correspondingly used an observation period of 3 months in their studies, 5 (14%) of them used a shorter observation period, and 5 (14%) used a longer one. In addition, 2 (6%) DiGAs suggested a use duration exceeding 3 months; 1 (3%) mirrored this with a longer study period, whereas the other did not. Two DiGAs advised a shorter use duration than 3 months and aligned their study duration accordingly.

The description of the study design varied widely among the 36 trials reviewed. A total of 13 (37%) DiGAs included detailed information with a link to a scientific publication in the study registry. Furthermore, 8 (23%) DiGAs provided a study report within the registry and 3 (9%) DiGAs published their study protocol. In addition, for 2 (6%) DiGAs, links to scientific publications were available in the DiGA directory.

**Figure 9:**
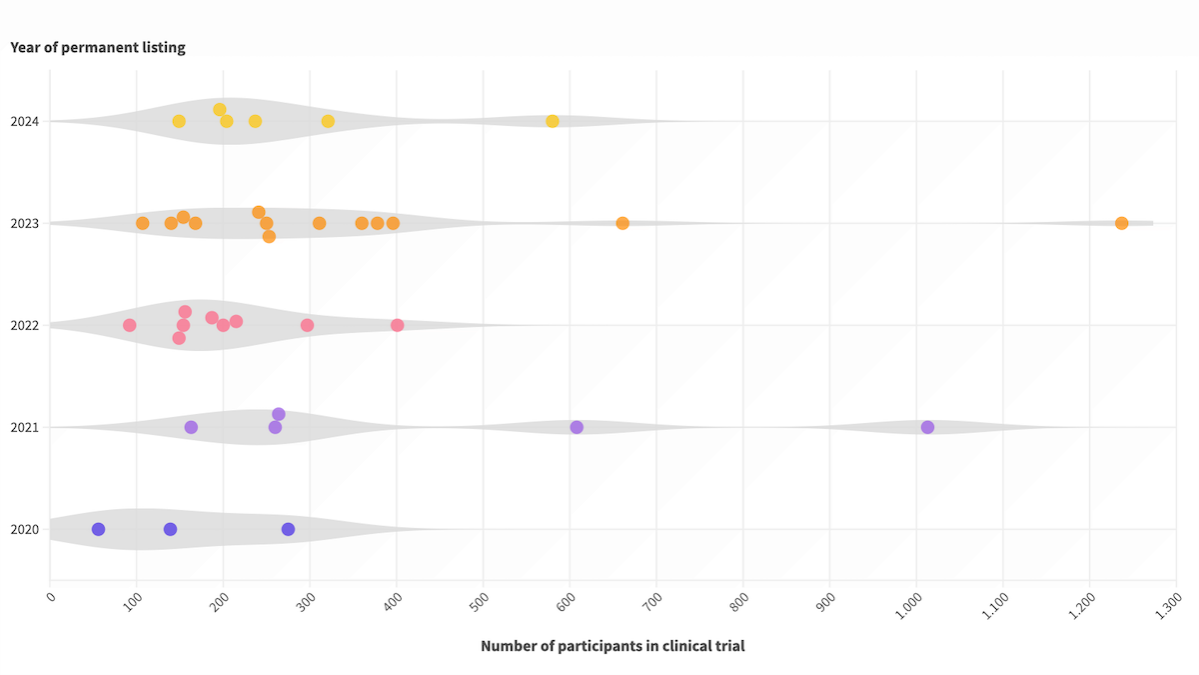
Year of permanent digital health application (Digitale Gesundheitsanwendung; DiGA) listing versus sample size in clinical trials (n=36).

For all 36 studies, the sample size information was provided, ranging from as low as 56 participants to as high as 1237, with the median being 239 participants. There has been a noticeable increase in sample size over the years: DiGAs that were permanently listed within the first 3 months in 2020 had a median of 139 (IQR 56-139) participants (combining both intervention and control groups), which rose to a median of 264 (IQR 211-810) participants in 2021, decreased slightly to 187 (IQR 151-256) in 2022, and then increased again to 253 (IQR 161-387) in 2023 and 220 (IQR 184-386) in 2024 (Figure 9).

Data on the duration of the clinical study were accessible for 30 trials, spanning from 4 months at the minimum to 54 months at the maximum, with a median duration of 17.5 months. Data on the dropout rate were available for 27 of the studies, indicating a median dropout rate of 17.5% among participants. Gender distribution was reported in 22 studies, revealing that approximately 63% of all study participants for which the gender was reported were female, 37% male, and less than 1% identified as diverse. The average age of participants, provided in 15 studies, had a median of 41.3 years. In comparison, the 2024 annual report of GKV-SV presented a gender distribution of 71% female and 29% male among actual DiGA users, with an average user age of 54 years.

## Discussion

### Principal Findings and Comparison With Prior Work

In summary, our results paint a picture of the emerging DiGA ecosystem in Germany. Through a data-driven examination, this paper uncovered the complexities and challenges in market dynamics, pricing strategies, prescription trends, and clinical evidence, offering valuable implications for researchers, DiGA manufacturers, regulatory bodies and health care professionals.

As of July 1, 2024, BfArM has approved 64 DiGAs, with 35 being permanently listed, 21 being preliminarily listed, and 8 that were subsequently removed from the directory. This study’s analysis, focusing on the 56 currently listed DiGAs, highlights the challenges in transitioning from preliminary to permanent listing. While 75% (24/32) of the DiGAs that were initially preliminary listed eventually achieved permanent listing, they often exceeded the 1-year timeline provided for this transition, with a third requiring exactly 2 years to complete the process. Although we do not have a rationale for this long timeframe in our data set, we anticipate that the process of evidence generation for digital medical devices is lengthy and that finding more appropriate methodologies for this purpose will require more academic attention in the future [4,6,22,23]. Our analysis reveals a wide therapeutic application of DiGAs across 12 categories, with psychological conditions being the most addressed, followed by endocrine and metabolic disorders. Despite the introduction of the positive care effect concept to encompass both medical benefits and patient-relevant structural and procedural improvements, the adoption of pSVV remains limited. Our economic analysis shows a steady year-on-year increase in both the initial and final prices of newly listed DiGAs. The appropriateness of these prices remains a topic of ongoing debate [24]. Looking ahead, the introduction of performance-based pricing models is anticipated to address these concerns by aligning costs more closely with the value and effectiveness of DiGAs [2,4].

Prescription analysis reveals a substantial, yet not overwhelming increase in DiGA use, attributed to both the introduction of new DiGAs to the market and the growth of existing DiGAs. Overall, the 15 most successful account for approximately 82% of total prescriptions. The year-on-year growth rate for DiGA prescriptions among the top 15 has shown a decline. Being first to market appears to be a critical factor for achieving substantial sales, although other elements, such as product quality, perceptions of health care professionals on DiGA [25,26], and the extent of sales efforts, obviously play vital roles but could not be analyzed in this study. While the current analysis has not yielded statistically significant results, the expanding market and the anticipated increase in the number of DiGAs suggest that statistically significant findings are likely to emerge in the near future.

The validity of study designs specifically for DiGAs remains a hotly debated topic [27]. The uniformity in choosing randomized controlled trials over alternative study designs for the primary clinical end point underscores an area ripe for exploration. Researchers are encouraged to investigate the impact of new study designs on the validation of digital health technologies [28,29], also with respect to continuous data evaluation [2]. Clinical trials for permanently listed DiGAs uniformly focused on medical benefits as positive care effect, with a universal emphasis on the *improvement of the state of health*. Thus, clinical trials for DiGAs so far adhere closely to the established methodologies common in the pharmaceutical sector. In the analysis of clinical studies, it was noted that the type and extent of data points made available to the public vary considerably. For example, although the gender ratio of study participants must be submitted to the BfArM for assessment, only 22 (61%) out of the 36 studies have made this information available to the public. In terms of advancing methodologies specifically for DiGAs, it would be beneficial to mandate the comprehensive publication of data points to be able to report relevant trends.

The modest uptake of pSVV as a novel regulatory concept highlights a gap in the market [30]. This calls for further research into the barriers to adoption and the potential of pSVV to broaden the scope of valued outcomes in digital health, potentially enhancing the effectiveness and patient-centeredness of DiGAs [7,31,32]. The hesitancy to adopt this new concept is also a reminder for regulatory bodies that regulatory innovation also requires accompanying measures during the introduction period.

With a median duration of 17.5 months for clinical trials, it logically follows that the initially targeted timeframe of 1 year from preliminary to permanent listing is not achievable for most manufacturers. However, our data also show that the fast-track procedure and preliminary listing of DiGA are a way of mitigating some of the challenges associated with randomized controlled trials [22,24,33]. Remarkably, 75% (24/32) of the DiGAs successfully transitioned from provisional to permanent listing. Furthermore, while more DiGAs will come to the market in the following years, this provides a fertile ground for analyzing DiGA’s therapeutic and economic impact together using a holistic model [34].

This paper not only sheds light on the current state of DiGA in Germany, but also signals areas for future investigation and strategic adjustment. By highlighting the need for diversified clinical study designs, a deeper exploration into regulatory innovations, such as pSVV and their adoptions, and holistic economic analysis, it offers a road map for enhancing the integration, effectiveness, and acceptance of digital health technologies within the German health care system and beyond.

### Implications for DiGA Manufacturers and Health Care Professionals

While causality cannot be definitively established, our findings suggest that DiGAs available as native apps generally see higher prescription numbers. Intriguingly, DiGA manufacturers with a larger portfolio in the market often offer web applications only, which tend to be less successful than those provided by manufacturers specializing on one or a few native apps. Having this in mind, DiGA manufacturers should consider developing native apps from the beginning. In addition, examining the implications of being first to market within specific *ICD-10* categories offers a unique lens through which to assess competitive advantage and market penetration strategies for digital health solutions. Considering that only 15 DiGAs have reached a milestone of 5000 prescriptions over their lifetime, it is crucial for DiGA manufacturers to recognize that listing in the DiGA directory does not guarantee automatic prescription success.

For health care professionals, it is essential to understand that DiGAs are subject to clinical trials akin to those required for pharmaceuticals. Moreover, the transition from preliminary to permanent listing fails for only a small number of DiGAs, highlighting the stringent evaluation process by the BfArM even at the initial listing stage. Despite public and media concerns about the potential risks associated with preliminarily listed DiGAs, our findings do not support the notion that these CE-certified, preliminarily listed DiGAs fail to deliver on their promised benefits. In addition, our findings indicate relatively positive user feedback on DiGAs, aligning with the extant research [17].

### Limitations and Further Research

The insights from this study are constrained by the limited public availability of detailed prescription data beyond the top 15 DiGAs and the variability in reporting standards across DiGA clinical trials. Obviously, individuals outside the statutory health insurance system and even those outside of Germany can purchase DiGAs privately. However, due to the absence of publicly accessible data on these transactions, we were unable to incorporate this information into our analysis.

Further health economics analyses, such as correlating prescription data with the actual number of affected individuals in Germany, would be valuable as well. However, conducting these comprehensive calculations requires a larger and more robust data set with respect to prescriptions. Similarly, due to the limited number of DiGAs currently available, statistically robust calculations were not feasible at this stage. As the market continues to expand, it is anticipated that such analyses will become possible in the future. Finally, we did not assess the quality and robustness of the clinical studies in our sample [7,35] as our goal was to map rather than clinically assess the emerging DiGA ecosystem.

Furthermore, we acknowledge that the presented findings do not address all discussions and criticisms that are being debated within the German statutory health care system. For instance, it is not possible, on the basis of currently available public data, to scientifically comment on the economic impact of DiGAs on various stakeholders and doubts regarding the actual uptake and adherence to DiGAs after prescription [11-13,36-38]. We anticipate that the introduction of performance-based pricing components from 2026 onward, aimed at measuring use, patient satisfaction, and patient health outcomes, may provide usable data for future research in this area. In addition, the integration of economic aspects into the approval and pricing process of DiGAs, like the benefit assessment for pharmaceuticals [39], could be considered in this context.

### Conclusions

As part of the 25th anniversary issue of the *Journal of Medical Internet Research*, this paper explores the thrilling early years of DiGAs in Germany, which marked an important stage in the digital transformation of health care. While DiGAs have not yet accelerated on a fast track to widespread adoption, their incremental positive influence on health care professionals and patients hints at a promising future. With their modest current uptake, there is a clear call for intensified efforts to boost their use in the coming years, ensuring that the best of digital health in Germany, Europe, and beyond is yet to be realized. The limited adoption of pSVV and the present monotony in clinical trial design are prominent areas for further research and potential regulatory attention.

It is encouraging to see that legislators have already adapted the DiGA fast-track procedure twice so far in 2024 (Digital Law) and in 2021 (Digital Modernization of Care and Nursing Act [German: *Digitale-Versorgung - und - Pflege - Modernisierungs - Gesetz*]) and has thus gradually adapted it in the spirit of agile legislation. Particularly noteworthy are the extended periods for permanent listing, the permission to also approve class IIb medical devices, and the fact that a path to performance–based pricing components has been introduced [4]. It is anticipated that further changes will be forthcoming, and this is a highly respectable approach that better reflects the reality of *digital* medical devices.

Enhancing transparency in DiGA use data and promoting methodological variety in clinical trials are crucial steps toward leveraging the full potential of digital health technologies for patient care. The insights provided by this study not only enrich our understanding of the DiGA landscape, but also underscore the necessity for continuous evaluation and adaptation within this fast-evolving sector. As DiGAs slowly start to make a positive impact, it is evident that the journey toward identifying and establishing study designs for digital health applications, which are broadly accepted across the market, is far from completion. The advancement of scientific dialogue is pivotal, serving as a crucial element in securing acceptance by both market participants and regulatory bodies.

## Appendix

Multimedia Appendix 1 Overview of clinical trials of all permanently listed digital health applications (Digitale Gesundheitsanwendung; DiGAs) as of July 1, 2024.

## Abbreviations

BfArM: Bundesinstitut für Arzneimittel und Medizinprodukte (Federal Institute for Drugs and Medical Devices)
DiGA: Digitale Gesundheitsanwendung (digital health application)
GKV-SV: National Association of Statutory Health Insurance Funds
ICD-10: International Classification of Diseases, Tenth Edition
MDD: Medical Device Directive
MDR: Medical Device Regulation
PHQ-9: Patient Health Questionnaire
pSVV: patientenrelevante Struktur- und Verfahrensverbesserung (patient-relevant improvement of structure and processes)
pVE: Positiver Versorgungseffekt (positive care effect)
