# Supplementary material for: Digital Health Applications (DiGAs) on a Fast Track: Insights From a Data-Driven Analysis of Prescribable Digital Therapeutics in Germany From 2020 to Mid-2024
Source: J Med Internet Res. 2024 Aug 29;26:e59013. doi: 10.2196/59013 (PMC11393499; doi:10.2196/59013)
Supplement: Multimedia Appendix 1 [file jmir_v26i1e59013_app1.pdf]

DiGA	Study Registry	Study ID	Publications
Cara Care für Reizdarm	DRKS	DRKS00026631	n/a
companion patella powered by medi - proved by Dt. Kniegesellsch	DRKS	DRKS00027261	n/a
deprexis - Studie 1	NCT	NCT01636752	Klein, J. P., Berger, T., Schröder, J., Späth, C., Meyer, B., Caspar, F., ... & Hohagen, F. (2016). Effects of a psychological internet intervention in the treatment of mild to moderate depressive symptoms: results of the EVIDENT study, a randomized controlled trial. *Psychotherapy and psychosomatics* , *85* (4), 218-228.
deprexis - Studie 2	NCT	NCT02178631	Klein, J. P., Berger, T., Schröder, J., Späth, C., Meyer, B., Caspar, F., ... & Moritz, S. (2013). The EVIDENT-trial: protocol and rationale of a multicenter randomized controlled trial testing the effectiveness of an online-based psychological intervention. *BMC psychiatry* , *13* , 1-10.
edupression	NCT / DRKS	NCT04839822 / DRKS00032121	n/a
elevida	ISRCTN	ISRCTN25692173	Pöttgen, J., Moss-Morris, R., Wendebourg, J. M., Feddersen, L., Lau, S., Köpke, S., ... & Gold, S. M. (2018). Randomised controlled trial of a self-guided online fatigue intervention in multiple sclerosis. *Journal of Neurology, Neurosurgery & Psychiatry* , *89* (9), 970-976.
Endo-App	NCT	NCT04883073	n/a
HelloBetter Diabetes und Depression	DRKS	DRKS00004748	Nobis, S., Lehr, D., Ebert, D. D., Baumeister, H., Snoek, F., Riper, H., & Berking, M. (2015). Efficacy of a web-based intervention with mobile phone support in treating depressive symptoms in adults with type 1 and type 2 diabetes: a randomized controlled trial. *Diabetes Care* , *38* (5), 776-783.
HelloBetter Panik	DRKS	DRKS00005223	Ebenfeld, L., Lehr, D., Ebert, D. D., Kleine Stegemann, S., Riper, H., Funk, B., & Berking, M. (2021). Evaluating a hybrid web-based training program for panic disorder and agoraphobia: randomized controlled trial. *Journal of medical Internet research* , *23* (3), e20829.
HelloBetter chronischer Schmerz	DRKS	DRKS00027176	n/a
HelloBetter Stress und Burnout	DRKS	DRKS00004749	Heber et al. (2013) - Efficacy and cost-effectiveness of a web-based and mobile stress management intervention for employees: design of a randomized controlled trialHeber at al. (2016) - Web-Based and Mobile Stress Management Intervention for Employees: A Randomized Controlled Trial
HelloBetter Vaginismus Plus	DRKS	DRKS00010228	Zarski, A. C., Berking, M., & Ebert, D. D. (2018). Efficacy of internet-based guided treatment for genito-pelvic pain/penetration disorder: rationale, treatment protocol, and design of a randomized controlled trial. *Frontiers in psychiatry* , *8* , 260.
Invirto- Die Therapie gegen Angst	NCT	NCT05510804	n/a
Kaia Rückenschmerzen - Rückentraining für Zuhause	DRKS	DRKS00015048	n/a
Kalmeda	DRKS	DRKS00022973	n/a
Kranus Edera	DRKS	DRKS00029182	Walter, U., Pennig, S., Kottmann, T., Bleckmann, L., Röschmann-Doose, K., & Schlee, W. (2023). Randomized controlled trial of a smartphone-based cognitive behavioral therapy for chronic tinnitus. PLOS Digital Health, 2(9), e0000337.
Kranus Lutera	DRKS	DRKS00030935	n/a
Mawendo	DRKS	DRKS00023454	n/a
Meine Tinnitus App - Das digitale Tinnitus Counseling	DRKS	DRKS00025379	n/a
Mindable: Panikstörung und Agoraphobie	DRKS	DRKS00029090	n/a
neolexon Aphasie	DRKS	DRKS00026233	n/a
NichtraucherHelden-App	DRKS	DRKS00025933	Rupp, A., Rietzler, S., Di Lellis, M. A., Weiland, T., Tschirner, C., & Kreuter, M. (2024). Digital smoking cessation with a comprehensive guideline-based app—results of a nationwide, multicentric, parallel, randomized controlled trial in Germany. *Nicotine and Tobacco Research* , *26* (7), 895-902.
Novego: Depressionen bewältigen	DRKS	DRKS00027459	n/a
Oviva Direkt für Adipositas	DRKS	DRKS00025291	n/a
PINK! Coach	DRKS	DRKS00028699	n/a
priovi - digitale Unterstützung der Borderline-Behandlung	DRKS	DRKS00028888	n/a
Selfapys Online-Kurs bei Binge-Eating-Störung	NCT	NCT04876183	Pruessner, L., Hartmann, S., Rubel, J. A., Lalk, C., Barnow, S., & Timm, C. (2022). Integrating a web-based intervention into routine care of binge-eating disorder: Study protocol for a randomized controlled trial. *Internet interventions* , *28* , 100514.
Selfapys Online-Kurs bei Bulimia Nervosa	NCT	NCT04876196	Hartmann, S., Pruessner, L., Rubel, J. A., Lalk, C., Barnow, S., & Timm, C. (2022). Applying a web-based self-help intervention for bulimia nervosa in routine care: Study protocol for a randomized controlled trial. *Internet Interventions* , *28* , 100512.
Selfapys Online-Kurs bei Depression	DRKS	DRKS00017191	Krämer, R., Köhne-Volland, L., Schumacher, A., & Köhler, S. (2022). Efficacy of a web-based intervention for depressive disorders: three-arm randomized controlled trial comparing guided and unguided self-help with waitlist control. *JMIR formative research* , *6* (4), e34330.
Selfapys Online-Kurs bei Generalisierter Angststörung	DRKS	DRKS00023799	n/a
somnio	NCT	NCT02629913	Lorenz, N., Heim, E., Roetger, A., Birrer, E., & Maercker, A. (2019). Randomized controlled trial to test the efficacy of an unguided online intervention with automated feedback for the treatment of insomnia. *Behavioural and cognitive psychotherapy* , *47* (3), 287-302.
velibra	ISRCTN	ISRCTN81412545	Berger, T., Urech, A., Krieger, T., Stolz, T., Schulz, A., Vincent, A., ... & Meyer, B. (2017). Effects of a transdiagnostic unguided Internet intervention ('velibra') for anxiety disorders in primary care: results of a randomized controlled trial. *Psychological medicine* , *47* (1), 67-80.
Vitadio	DRKS	DRKS00027405	n/a
Vivira	DRKS	DRKS00022781	Weise, H., Zenner, B., Schmiedchen, B., Benning, L., Bulitta, M., Schmitz, D., & Weise, K. (2022). The effect of an app-based home exercise program on self-reported pain intensity in unspecific and degenerative back pain: pragmatic open-label randomized controlled trial. *Journal of medical Internet research* , *24* (10), e41899.
vorvida	DRKS	DRKS00006104	Zill, J. M., Christalle, E., Meyer, B., Härter, M., & Dirmaier, J. (2019). The effectiveness of an Internet intervention aimed at reducing alcohol consumption in adults: Results of a randomized controlled trial (Vorvida). *Deutsches Ärzteblatt International* , *116* (8), 127.
zanadio	DRKS	DRKS00024415	Roth, L., Ordnung, M., Forkmann, K., Mehl, N., & Horstmann, A. (2023). A randomized-controlled trial to evaluate the app-based multimodal weight loss program zanadio for patients with obesity. *Obesity* , *31* (5), 1300-1310.

This Mulimedia Appendix is associated to the article:
Goeldner M & Gehder S: Digital Health Applications (DiGAs) on a Fast Track: Insights From a Data-Driven Analysis of Prescribable Digital Therapeutics in Germany From 2020 to Mid-2024
J Med Internet Res 2024;26:e59013
URL: https://www.jmir.org/2024/1/e59013/
doi: 10.2196/59013
Data as of July 1st, 2024. Contact: www.tuhh.de/ddi
